# Autophagy and Cellular Senescence Mediated by Sox2 Suppress Malignancy of Cancer Cells

**DOI:** 10.1371/journal.pone.0057172

**Published:** 2013-02-25

**Authors:** Yong-Yeon Cho, Dong Joon Kim, Hye Suk Lee, Chul-Ho Jeong, Eun-Jin Cho, Myong-Ok Kim, Sanguine Byun, Kun-Yeong Lee, Ke Yao, Andria Carper, Alyssa Langfald, Ann M. Bode, Zigang Dong

**Affiliations:** 1 The Hormel Institute, University of Minnesota, Austin, Minnesota, United States of America; 2 College of Pharmacy, The Catholic University of Korea, Gyeonggi-do, Korea; Cedars-Sinai Medical Center, United States of America

## Abstract

Autophagy is a critical cellular process required for maintaining cellular homeostasis in health and disease states, but the molecular mechanisms and impact of autophagy on cancer is not fully understood. Here, we found that Sox2, a key transcription factor in the regulation of the “stemness” of embryonic stem cells and induced-pluripotent stem cells, strongly induced autophagic phenomena, including intracellular vacuole formation and lysosomal activation in colon cancer cells. The activation occurred through Sox2-mediated *ATG10* gene expression and resulted in the inhibition of cell proliferation and anchorage-independent colony growth *ex vivo* and tumor growth *in vivo.* Further, we found that Sox2-induced-autophagy enhanced cellular senescence by up-regulating tumor suppressors or senescence factors, including p16^INK4a^, p21 and phosphorylated p53 (Ser15). Notably, knockdown of *ATG10* in *Sox2*-expressing colon cancer cells restored cancer cell properties. Taken together, our results demonstrated that regulation of autophagy mediated by Sox2 is a mechanism-driven novel strategy to treat human colon cancers.

## Introduction

Cancer cells, but not normal cells, acquire immortalization by escaping cellular senescence [1]. Compared to normal cells, cancer cells usually require more nutrition to produce proteins and enzymes for their faster proliferation, which results in nutritional dependence of cancer cells [2]. Survival is supported by nutrients obtained through the blood supply and by self digestion of intracellular organelles in a process referred to as autophagy [3].

Autophagy is a conserved catabolic process in eukaryotes that degrades long-lived proteins, organelles, and bulk cytoplasm [4] through the lysosomal system to maintain homeostasis during starvation and normal growth control [3]. Autophagy promotes cell survival by purging the cells of damaged organelles, toxic metabolites, and intracellular pathogens and by generating the intracellular building blocks required to maintain vital functions during nutrient-limited conditions [5]. However, autophagy might also comprise an important strategy to treat cancer because autophagy can also promote cell death through excessive self-digestion and degradation of essential cellular constituents [5]. Autophagy is different from apoptosis, a process that lacks autophagosomes and autolysosomes in dying cells [6]. Notably, blockage of autophagy by knocking out beclin induces tumor development [7] and inducing autophagy by treatment with natural compounds, such as resveratrol, suppresses cancer cell proliferation and malignancy [2]. Autophagy is reportedly closely related with cellular senescence and inhibition of autophagy delays cellular senescence in mitotic human diploid cells [8]. These observations indicate that autophagy might induce senescence in cancer cells because inhibition of autophagy enhances tumorigenic properties of MCF-7 cells [9]. However, detailed mechanisms have not been clearly elucidated.

Furthermore, tamoxifen, an antagonist of the estrogen receptor in breast tissue, induces autophagy in human MCF-7 breast cancer cells [10] mediated through sphingolipid metabolites [11]. Notably, arsenic trioxide, imatinib (Gleevec) and rapamycin are compounds known to induce autophagic cell death in many human cancer cell lines [12], [13], [14]. Importantly, ovary, breast and prostate cancers are associated with monoallelic loss of beclin1 in humans. Furthermore, induction of autophagy by beclin overexpression suppresses anchorage-independent colony growth of MCF-7 cells [15] and proliferation by negative inhibition of p70S6 kinase [16], [17].

Both classical and modernized cellular reprogramming are stressful processes that activate apoptosis and cellular senescence, which are the two primary barriers to cancer development and somatic reprogramming [18]. The mTOR signaling pathway shares in the reprogramming of somatic cells, cellular senescence and autophagy formation [18]. For example, well-characterized mTOR inhibitors and autophagy activators, including PP242, rapamycin and resveratrol, notably improve the speed and efficiency of iPS cell generation [19]. Sox2, a transcription factor belonging to the HMG (High Mobility Group) superfamily, has a role in determination of cell fate, differentiation and proliferation [20]. It is also a key regulator of embryonic stem (ES) cell self-renewal and reprogramming of terminally differentiated cells to iPS cells [21]. Although the oncogenic potential of stem cell factors, such as Sox2, has been hypothesized to be based on the “stemness” similarity between stem cells and cancer cells, Sox2’s function in cancer cells is not clearly understood. Furthermore, recent studies have shown that the Sox2 protein level strongly corresponds with less differentiated basal cell-like breast carcinomas [22], and the Sox2 protein is frequently found to be down-regulated in gastric carcinomas [23]. These types of studies have caused more controversy regarding the normal function and oncogenic potential of stem cell factors. Here, we provide evidence showing that expression of Sox2 in cancer cells induces autophagy of cancer cells by up-regulating *ATG10* gene expression and inducing cellular senescence, resulting in reduced malignancy of cancer cells and inhibition of tumor growth *ex vivo* and *in vivo*.

## Results

### Ectopic Expression of *Sox2* Induces Autophagy

**Figure 1:**
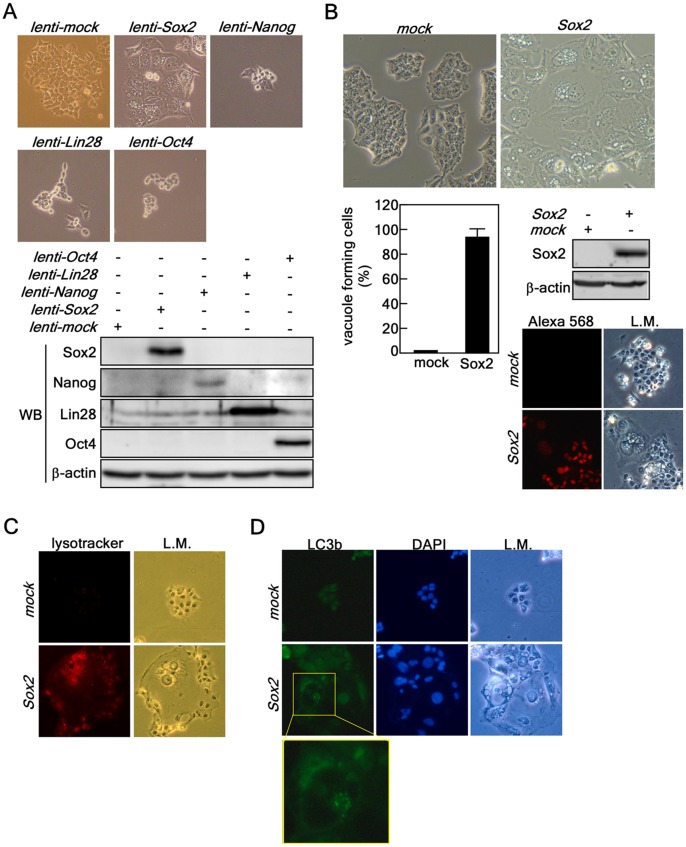
Ectopic expression of Sox2 induces autophagy. (*A*) Comparison of morphological changes induced by ectopic expression of iPS factors. *(Upper panels)* HCT116 cells were individually transduced with iPS factors, including Sox2, Nanog, Lin28 and Oct4. The cells were cultured for 5 days and changes were observed under a light microscope (X200). *(Lower panels)* HCT116 cells were harvested at 5 days after transduction with iPS factors and proteins extracted. The protein levels of the Sox2, Nanog, Lin28 and Oct4 were analyzed by Western blotting with specific antibodies as indicated. ß-Actin was used as an internal control to verify equal protein loading. (*B*) HCT116 cells forming vacuoles at 5 days after transduction were observed under light microscopy, counted and compared. Sox2 overexpression was analyzed by Western blotting and immunocytofluorescence assay (X200) using specific antibodies as indicated. ß-Actin was used as an internal control to verify equal protein loading. The cells were visualized by light microscopy (L.M.; X200). (*C*) Lysosomal activation analysis. HCT116 cells infected with *mock* or *Sox2* were stained by adding lysotracker (50 nM) into the culture medium for 5 min in a 37 ^o^C, 5% CO_2_ incubator. The cells were fixed with 4% formalin, washed with PBS and lysosomal activation was observed under a fluorescence microscope (X200). (*D*) Immunofluorescence assay of LC3b (i.e., ATG8b). HCT116 cells stably expressing *mock* or *Sox2* were subjected to a fluorescence assay to detect LC3b. The cells were observed under a fluorescence microscope (X200). The nuclei were stained with DAPI; L.M. indicates light microscopy (X200).

Recent studies indicated that ectopic expression of *Sox2* by retroviral infection into MCF-7 breast cancer cells increased both the size and number of colonies formed in soft agar [24]. However, Sox2 is frequently down-regulated in gastric cancers and inhibits cell growth through cell cycle arrest and apoptosis [23]. Therefore, the role of Sox2 in cancer is controversial. To explore the role of Sox2 and other iPS factors in cancer, we ectopically expressed these factors in HCT116 human colorectal cancer cells and found that Sox2, but not Nanog, Lin28 or Oct4, induced severe vacuole formation in the cytoplasm, which is an important marker of macroautophagy [25] (**Fig. 1A**). We found that over 90% of infected cells formed different sized vacuoles in their cytoplasm and Western blotting and immunocytofluorescence assay results indicated that all the cells expressed the ectopic Sox2 protein (**Fig. 1B**). Further, we confirmed that severe vacuole formation coincided with acidic lysosomal activation in HCT116 colon cancer cells (**Fig. 1C**). Importantly, Sox2 overexpression induced LC3 (also known as ATG8b) foci formation, which is a key biomarker of autophagy (**Fig. 1D**). These results indicated that Sox2 overexpression induced autophagy.

### Sox2 Induces Autophagy in Cancer Cells, but not in Normal Cells

**Figure 2:**
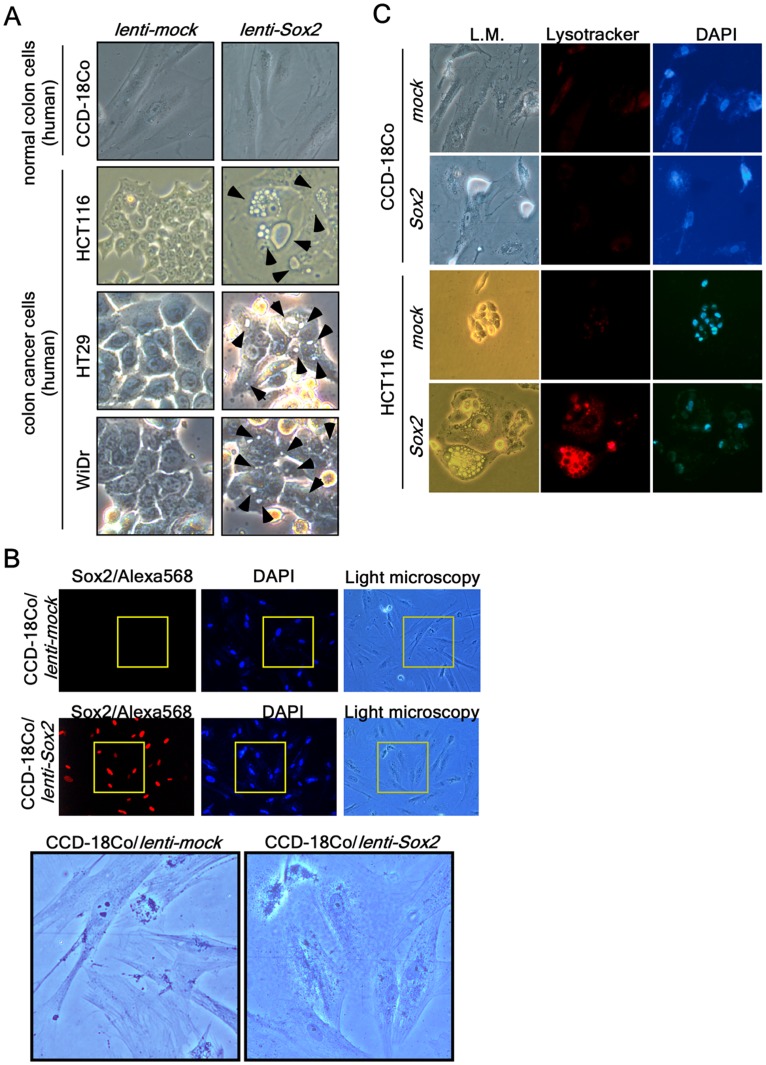
Cancer cell-specific lysosomal activation. (*A*) CCD-18Co (CRL-1459) normal colon cells and HCT116, HT29 and WiDr colon cancer cells were cultured in the appropriate medium and transduced with *Sox2* viral particles. The cells were cultured with complete growth medium for 5 days after transduction. The cells were observed under a light microscope. Cellular vacuoles are marked with an arrowhead in *Sox2*-expressing colon cancer cells. (*B*) CCD-18Co normal colon cells were cultured, transduced with *mock* or *Sox2* viral particles and cultured with complete growth medium for 5 days. The cells were then fixed, permeabilized and hybridized with a Sox2 specific antibody and then with an Alexa 568-conjugated secondary antibody, and visualized with a fluorescence microscope (X200). Nuclei were stained with DAPI. The areas of light microscopy are the areas matched with Sox2 and DAPI fluorescence. The boxed area under low power is magnified to compare the morphology between HCT116-*mock* and -*Sox2*-expressing cells. (*C*) Lysosomal activation, a autophagy marker, was compared by adding lysotracker-Red (50 nM) at 5 days after transduction to CCD-18Co normal colon cells and HCT116 colorectal cancer cells infected with *mock* or *Sox2*. The cells were observed under a fluorescence microscope. L.M. indicates the same area of light microscopy corresponding to fluorescence microscopy (X200).

To investigate whether Sox2 overexpression can induce vacuole formation in different colon cancer cell lines, we transduced lenti-Sox2 viral particles into CCD-18Co normal colon cells and HCT116, HT29 and WiDr human colon cancer cells. We found that all the colon cancer cell lines formed vacuoles in their cytoplasm (**Fig. 2A** **, arrows**). However, although CCD8-18Co normal colon cells showed good expression of Sox2 after transduction with lenti-Sox2, the cells did not form vacuoles or display morphological changes (**Fig. 2A, B**). Further, additional results confirmed that vacuole formation and acidic lysosomal activation were observed in HCT116 colon cancer cells, but not in CCD-18Co normal colon cells (**Fig. 2C**). In addition, ectopic expression of Sox2 in normal mouse embryonic fibroblasts (MEFs) or human primary fibroblasts (NFDH and BJ) did not cause vacuole formation (**data not shown**), demonstrating that vacuole formation induced by Sox2 overexpression in HCT116 cells is indeed cancer cell-specific autophagy.

### Sox2 Targets ATG10 to Induce Autophagy

**Figure 3:**
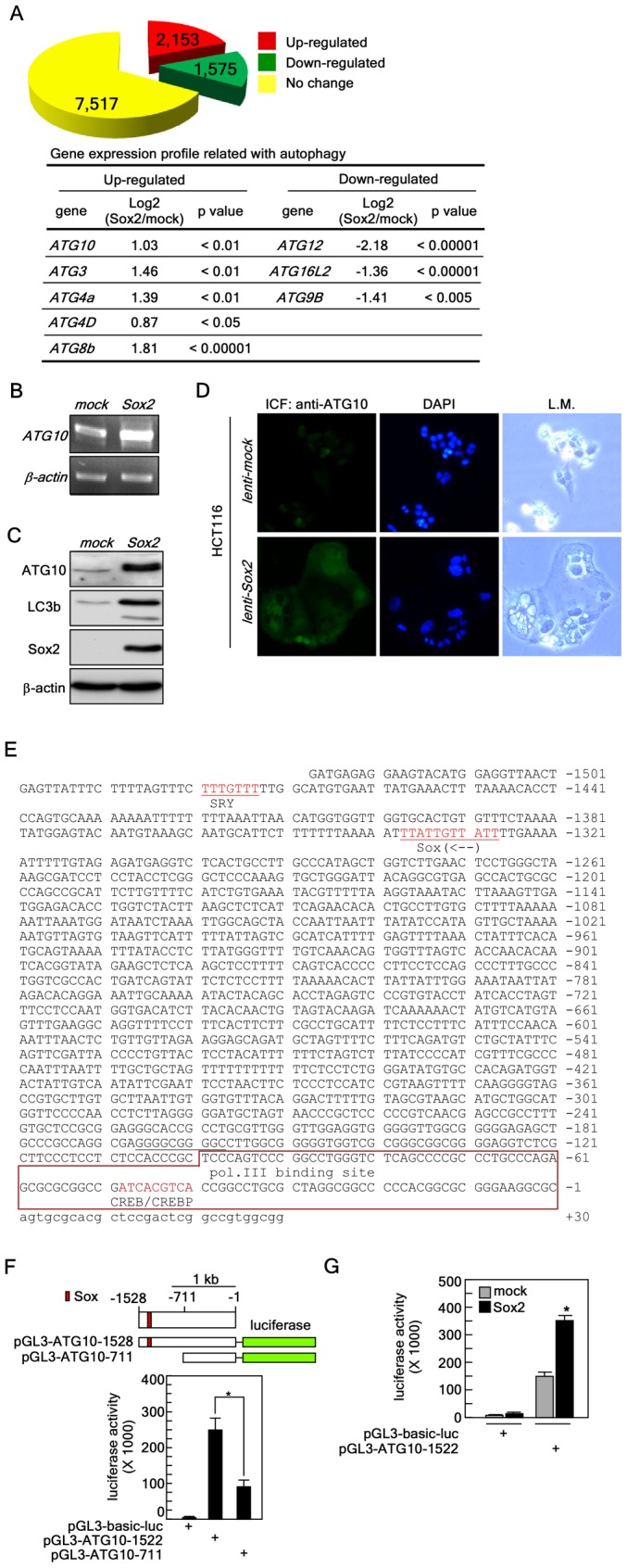
Sox2 targets ATG10 to induce autophagy. (*A*) Microarray. *Top panel*, HCT116 colorectal cancer cells stably expressing *mock* or *Sox2* were subjected to microarray analysis as described in “Materials and Methods”. The yellow color indicates the number of genes that did not show a significant difference between *mock* and *Sox2* expression. The red and green colors indicate the number of genes up- or down-regulated, respectively, in HCT116 cells stably expressing *Sox2* compared with cells expressing the *mock* control. *Bottom table*, summary of autophagy-related genes obtained from the microarray analysis. Up- and down-regulation are denoted in Log2 values. (*B*) Expression of *ATG10* induced by Sox2. Total RNA (1 µg) from HCT116 cells infected with *mock* or *Sox2* was reverse transcribed and *ATG10* was amplified by cycle-dependent PCR and the *ATG10* expression level was visualized by agarose gel electrophoresis at the 21^st^ cycle. ß-Actin was used as an internal control to verify equal utilization of cDNA for PCR. (*C*) Up-regulation of ATG10 and LC3b protein levels induced by *Sox2* expression. Proteins were extracted from HCT116 cells stably expressing *mock* or *Sox2* and ATG10 and LC3b proteins were visualized by Western blotting using specific antibodies. ß-Actin was used as an internal control to verify equal protein loading. (*D*) Confirmation of Sox2-induced ATG10 protein level. HCT116 colorectal cancer cells were infected with *mock* or *Sox2* and cultured 5 days. The cells were fixed, hybridized with an ATG10 specific primary antibody and Alexa 488-conjugated secondary antibody. The ATG10 protein levels were observed by fluorescence microscopy. L.M. indicates the same area of light microscopy corresponding to fluorescence microscopy (X200). (*E*) Construction of the *ATG10* promoter *luciferase* reporter plasmid. The nucleotide sequences of the human *ATG10* promoter region were downloaded from Ensemble (http://uswest.ensemble.org). The putative promoter analysis was conducted using TFSEARCH (v1.3) (http://cbrc.jp/htbin/nph-tfsearch). The putative SRY and Sox binding consensus sequences are denoted in red and the polymerase III binding site is boxed. (*F*) The BAC clone (RP11-111B20) was purchased from Empire Genomics (Buffalo, NY) and 1528 and 711 bp of the *ATG10* promoter region were amplified by PCR. The PCR fragment was recombined with the *pGL3* basic vector to construct pGL3-AT10-1582 and pGL3-ATG10-711 *luciferase* reporter plasmids. The *pGL3-ATG10-luc* reporter plasmids were confirmed by DNA sequencing. The *ATG10* promoter *luciferase* reporter plasmids, *pGL3-AT10-1582* and *pGL3-ATG10-71-*luc, were transiently transfected into HCT116 cells and the firefly luciferase activity was analyzed after 24 h. The *phRL-SV40 renilla* luciferase reporter plasmid was co-transfected as an internal control to verify equal transfection and normalization of firefly luciferase activity (*p<0.001). (*G*) The *pGL3-ATG10-1528* luciferase reporter plasmid was transiently co-transfected with a *mock* or *Sox2* viral expression vector into HCT116 cells and the firefly luciferase activity was analyzed after 24 h. The *phRL-SV40 renilla* luciferase reporter plasmid was co-transfected as an internal control to verify equal transfection and normalization of firefly luciferase activity (*p<0.001).

To explore the mechanism(s) of Sox2-induced autophagy, we first used a microarray analysis of a total of 30,968 genes from cDNAs isolated from cells infected with *mock* or *Sox2* to identify gene(s) targeted by Sox2 to induce autophagy. The results revealed 11,245 genes that were analyzed using the Significant Analysis of Microarray (SAM) program (http://www-stat.stanford.edu/~tibs/SAM). We found that 2,153 Sox2-induced genes could be classified as up-regulated and 1,575 genes were down-regulated in HCT116 cells (**Fig. 3A** **and Table S1**). We used the Database for Annotation, Visualization and Integrated Discovery (DAVID v6.7; http://niaid.abcc.ncifcrf.gove) to further classify the genes according to their biological or molecular functions and found that gene expression levels associated with autophagy, proliferation and cell cycle regulation were substantially altered by the ectopic expression of *Sox2.* Altered gene expression included changes in 33 DNA repair genes, 25 DNA replication genes, 20 cell growth-related genes, 26 cell size-related genes, 191 transcription-related genes and 17 insulin signaling pathway-related genes (**Fig. 3A** and **Tables S1, S2 and S3**). Importantly, *Sox2* expression induced increased expression of *ATG10, ATG3, ATG4a, ATG4D* and *ATG8b* genes by about 2–4 fold (**Fig. 3A**). In contrast, *Sox2* expression was associated with decreased expression of the *ATG12, ATG16L2* and *ATG9B* genes (**Fig. 3A**). By searching a database containing the Sox2 consensus-binding motif in the promoter region in the genome [26], we found that the *ATG10* and *ATG12* promoters contain a putative Sox2 binding consensus nucleotide motif harboring 63% identity (**Table S4**). Comparing our microarray and the database search results, we concluded that ATG10 might be a target of Sox2 in the induction of autophagy. Our cycle-dependent RT-PCR results demonstrated that the *ATG10* mRNA level from *Sox2-*transfected cells was increased by about 2.5 fold compared with *mock* (**Fig. 3B**). Notably, Western blotting results (**Fig. 3C**) and immunocytofluorescence against ATG10 (**Fig. 3D**) indicated that ATG10 and LC3 protein levels were increased by overexpression of Sox2. To determine whether the *ATG10* promoter is activated by Sox2, we constructed two *luciferase* reporter plasmids containing −1522 to −1 (*pGL3-ATG10-1522*) and −711 to −1 (*pGL3-ATG10-711*) (**Fig. 3E**) by searching and analyzing the *ATG10* promoter regions, which contain putative Sox and SRY binding motifs at −1327 and −1473 from the transcription initiation site (**Fig. 3E**). We found that that deletion of putative Sox2 binding region significantly suppressed luciferase activity (**Fig. 3F**). Notably, *ATG10* promoter activity was increased by overexpression of Sox2 (**Fig. 3G**), indicating that Sox2 induces *AT10* gene expression and causes autophagy.

### Sox2-induced Autophagy is Mediated Through the Down-regulation of the Akt Signaling Pathway, but not Through Class III PI3-K Signaling

**Figure 4:**
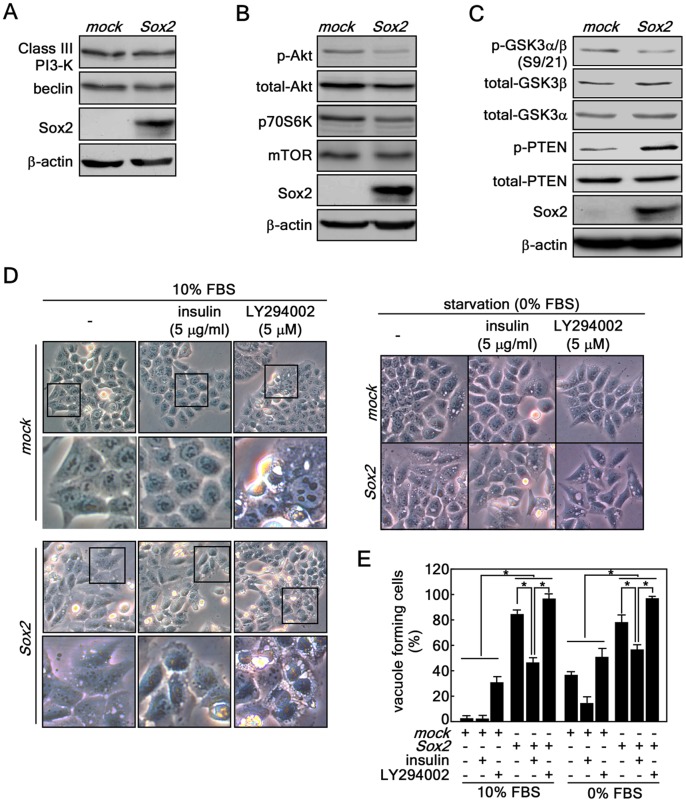
Sox2-induced autophagy is mediated through the down-regulation of the Akt signaling pathway, but not through Class III PI3-K signaling. (*A–C*) Protein levels of Class I signaling molecules including Akt, mTOR and p70S6K and class III PI3-K signaling molecules including Vps34p and beclin were analyzed in HCT116 cells stably expressing *mock* or *Sox2*. The individual proteins were visualized by Western blotting using specific antibodies. β-Actin was used as an internal control to verify equal protein loading. (*D*) Involvement of Class I PI3-K signaling in Sox2-induced autophagy. (*D, left panels*) HCT116 cells stably expressing *mock* or *Sox2* were treated with insulin or LY294002 under 10% FBS-containing conditions at 2 days after infection. Vacuole formation was observed by light microscopy (X200). The boxed areas are individually magnified 2 more times (*lower panels for mock and Sox2*) to better visualize vacuoles. *(D, right panels)* The cells were transduced with *Sox2* viral particles and cultured 2 days. The cell culture medium was replaced with McCoy’s 5a without FBS supplementation but including 5 µg/ml insulin or 5 µg LY294002 and then cells were cultured for 5 days. The cells were observed under light microscopy (X200). (*E*) Quantitative comparison of autophagy formation induced by class I PI3-K signaling. The vacuole forming cells from D and F were counted and compared in a numerate graphic (*p<0.001).

Fasting or depletion of growth factors such as insulin induces autophagy [27]. Our microarray results demonstrated that gene expression associated with the insulin signaling pathway was suppressed (**Table S3**). Therefore, we examined changes in protein levels associated with autophagy and insulin signaling by Western blotting. We found that Class III PI3-K (Vps34p) and beclin were not changed, indicating that the Class III PI3-K/beclin signaling pathway is not involved in Sox2-induced autophagy (**Fig. 4A**). By investigating the PI3-K-Akt signaling pathway, we found that Akt phosphorylation (Ser308) was substantially inhibited and the total protein levels of Akt, mTOR and p70S6K were decreased in *Sox2*-infected cells compared with *mock*-infected cells (**Fig. 4B**). To confirm signaling related to the inhibition of the PI3-K-Akt signaling pathway, we analyzed GSK3α/ß and PTEN. The results indicated that phosphorylation of GSK3α/ß at Ser9/Ser21 was suppressed and total GSK3α/ß protein levels were slightly increased in *Sox2*-infected cells compared with *mock*-infected cells (**Fig. 4C**). Notably, we found that PTEN phosphorylation was also increased (**Fig. 4C**), resulting in suppression of PI3-K signaling by de-phosphorylation [28]. These results indicated that Sox2 alters the PI3-K-Akt signaling pathway through GSK3α/ß and PTEN signaling, although the upstream specific kinase responsible for phosphorylation of PTEN is not known in this instance. We treated HCT116 cells expressing *Sox2* or *mock* with insulin or LY294002, a PI3-K inhibitor. Results indicated that *Sox2*-induced vacuole formation was markedly inhibited by insulin treatment (**Fig. 4D** *left panel* *s,* **E**). However, the *mock*-infected cells did not show vacuole formation whether or not treated with insulin in normal cell culture condition (10% FBS) or suppressed vacuole formation in starvation condition (0% FBS) (**Fig. 4D** *left and middle panels,* **E, F**). We also found that *mock*-infected cells exhibited increased vacuole formation after treatment with LY294002, whereas *Sox2* overexpression caused more vacuole formation induced by LY294002 treatment compared with *mock* (**Fig. 4D** *right panel* *s,* **E, F**). Similar phenomena were observed under starvation conditions (**Fig. 4E, F**). Taken together, these results indicated that the down-regulation of the PI3-K-Akt pathway by PTEN might cooperate to induce autophagy associated with Sox2 expression.

### Sox2-induced Autophagy Suppresses Proliferation and Anchorage-independent Colony Growth by Inducing Cellular Senescence in HCT116 Cells

**Figure 5:**
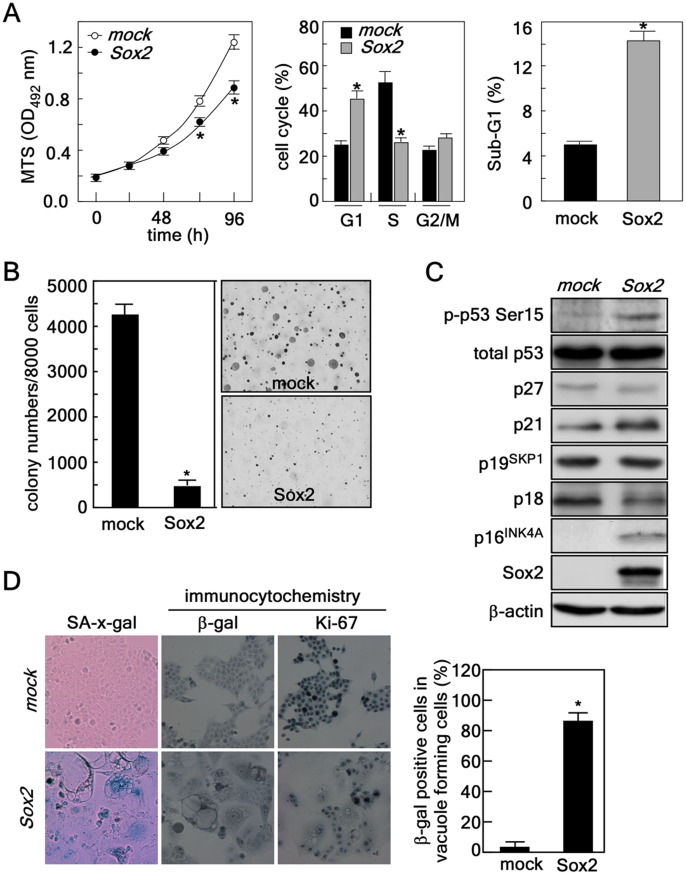
Sox2-induced autophagy causes senescence in HCT116 colorectal cancer cells resulting in suppressed proliferation and decreased anchorage-independent colony growth. (*A*) *Left panel*, effect of *Sox2* expression on cell proliferation. HCT116 cells (2×10^3^) stably expressing *mock* or *Sox2* were seeded in 96-well plates and proliferation was analyzed by MTS assay at 24 h intervals up to 96 h (*, p<0.001). *Middle and right panels*, effect of *Sox2* expression on cell cycle distribution. HCT116 cells (4×10^5^) stably expressing *mock* or *Sox2* were seeded, cultured and then cell cycle distribution (*middle panel*) and sub-G1 accumulation (*right panel*) were analyzed by FACS (*, p<0.001). (*B*) Effect of *Sox2* expression on anchorage-independent colony growth. HCT116 cells (8×10^3^) stably expressing *mock* or *Sox2* were subjected to a soft agar colony growth assay for 5–7 days. The cell colonies were scored using a microscope and the Image-Pro PLUS (v.6) computer software program (*p<0.001). (*C*) Protein profiling related to cell cycle regulation. Proteins were extracted from HCT116 cells stably expressing *mock* or *Sox2* and subjected to Western blotting to detect the level of various proteins known to be involved in cell cycle regulation. ß-Actin was used as an internal control to verify equal protein loading. (*D*) Cellular senescence assay. *Left panel*, HCT116 cells stably expressing *mock* or *Sox2* were analyzed for senescence using a senescence ß-galactosidase staining kit. The cells were observed under a light microscope (X200). *Middle* and *right panels*, the protein level of ß-galactosidase or Ki-67 was detected in HCT116 cells stably expressing *mock* or *Sox2* using specific primary antibodies as indicated and an HRP-conjugated secondary antibody. Immunocytochemical detection was performed using the Sigma FASTTM 3,3′-Diaminobenzidine Terahyfrochloride with Metal Enhancer Tablet Sets (DAB peroxidase substrate). The protein levels were visualized using a light microscope (X200). *Graph*, indicates a comparison of ß-galactosidase positive cell population in HCT116 cells stably expressing *mock* or *Sox2* (*p<0.001).

Suppression of PI3-K activity by PTEN has an important role in cell proliferation and cancer development in the colon [29], [30], [31]. PI3-K inhibition by PTEN or wortmannin has an inverse effect compared with tumor necrosis factor- α on the balance between the p50 and p65 subunits of nuclear factor *k*B [31]. GM3, a ganglioside, treatment dramatically increases cyclin-dependent kinase (CDK) inhibitor (CKI) p21^WAF1^ expression through the accumulation of the p53 protein by the PTEN-mediated inhibition of PI3-K-Akt-MDM2 survival signaling in HCT116 colon cancer cells [29]. In human colorectal cancer, a negative correlation between PTEN protein levels and Akt phosphorylation has been observed [30]. Our results indicated that down-regulation of PI3-K-Akt signaling through PTEN might be involved in Sox2-induced autophagy (**Fig. 4**). Our cell proliferation and cell cycle analyses indicated that *Sox2* infection into HCT116 cells suppressed proliferation by inducing accumulation of cells in G0/G1 and sub-G1 and reduction of S cell cycle phases compared with *mock*-infected cells (**Fig. 5A**). The attenuation of cell proliferation was associated with about 90% inhibition of anchorage-independent cancer growth in soft agar, a hallmark of cancer cell properties [32] (**Fig. 5B**). To explore the signaling responsible for inducing the loss of cancer cell properties such as suppression of proliferation and soft agar growth, we examined various tumor suppressors with a known role in regulating cell cycle. We observed an increase in p53 phosphorylation (Ser15) and increases in the total protein levels of p16^INK4a^ and p21 (**Fig. 5C**), which are well known to be strongly involved in cellular senescence [33]. We then examined senescence using an SA-ß-galactosidase assay and found that ectopic expression of *Sox2* enhanced ß-galactosidase activity and protein level (**Fig. 5D**, *left and middle panels*) and suppressed Ki-67 protein level, a cell proliferation marker (**Fig. 5D**, *right panel*). Notably, over 90% of cells containing vacuoles stained ß-gal positive (**Fig. 5D**, *graph*), Taken together, these results indicated that Sox2-induced autophagy causes a loss of tumor properties in HCT116 colorectal cancer cells.

### Knockdown of *ATG10* Restores *Sox2*-induced Autophagy, Cellular Senescence and Cell Proliferation

**Figure 6:**
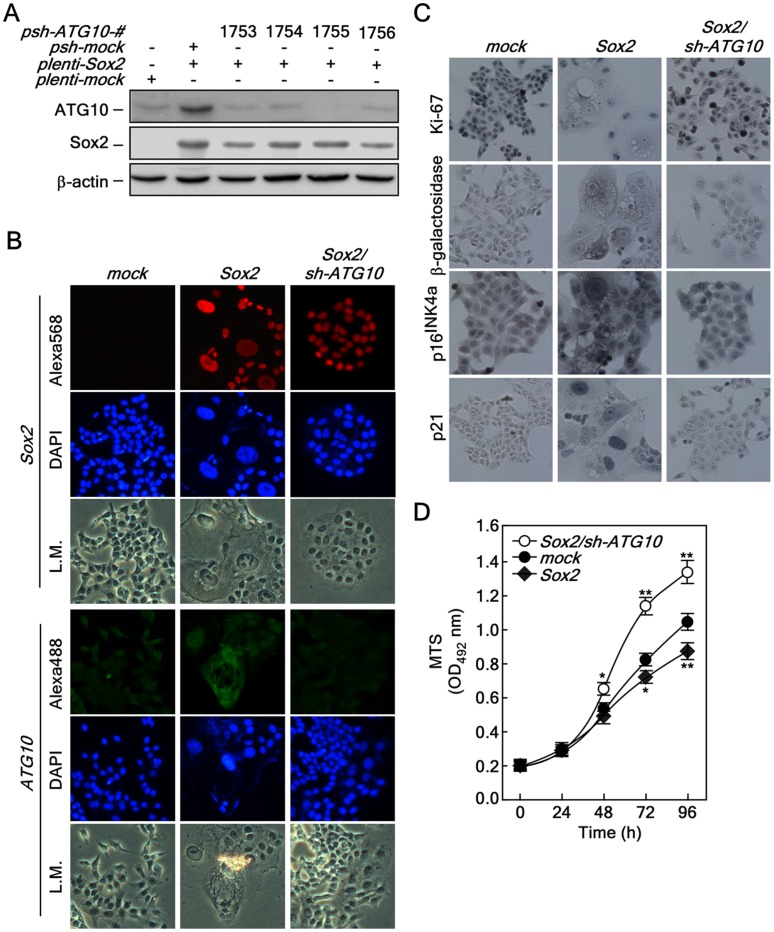
Knockdown of *ATG10* restores Sox2-induced autophagy, cellular senescence and proliferation. (*A*) Confirmation of the knockdown efficiency of ATG10. Knockdown of ATG10 using *pLKO-shATG10*. The *pLKO-shATG10* knockdown lenti-viral particles were infected into HCT116 cells stably expressing *Sox2* and cells cultured for 36 h. The proteins were extracted and knockdown efficiency was determined by Western blot using a specific ATG10 antibody. ß-Actin was used as an internal control to verify equal protein loading. (*B*) Morphological changes induced by *ATG10* knockdown in HCT116 cells stably expressing *Sox2*. HCT116-mock, -Sox2 and -Sox2/sh-ATG10 cells were analyzed for morphological changes and protein levels of Sox2 (red) and ATG10 (green) were determined by an immunofluorescence assay using specific antibodies. The cells were observed under a fluorescence or light microscope (X200). L.M. indicates light microscopy. (*C*) Restoration of cell cycle, cell proliferation and senescence markers by knocking down *ATG10* in HCT116 cells stably expressing *Sox2*. HCT116-mock, -Sox2 and -Sox2/sh-ATG10 cells were seeded into a 4-chamber slide, fixed and permeabilized. Markers for cell proliferation, cellular senescence and cell cycle regulation included Ki-67, ß-galactosidase, p16^INK4a^ and p21. These markers were analyzed by immunocytochemistry with specific antibodies as indicated using the Sigma FASTTM 3,3′-Diaminobenzidine Terahyfrochloride with Metal Enhancer Tablet Sets (DAB peroxidase substrate). The cells were observed under a fluorescence or light microscope (X200). (*D*) Restoration of proliferation by knocking down *ATG10* in HCT116 cells stably expressing *Sox2*. HCT116-mock, -Sox2 and -Sox2/sh-ATG10 cells (2×10^3^) were seeded into 96-well plates and proliferation was analyzed by MTS assay at 24 h intervals up to 96 h (*p<0.05, **p<0.001).

Our previous results demonstrated that *Sox2*-mediated autophagy is mediated through *ATG10* expression (**Fig. 3**). To examine whether knockdown of *ATG10* can suppress autophagy induced by Sox2 overexpression, we selected a *psh-ATG10-1755* knockdown vector (**Fig. 6A**). The *sh-ATG10* viral particles were infected into *Sox2*-preinfected cells and expression of Sox2 (**Fig. 6B**, *upper panels*) and knockdown of *ATG10* (**Fig. 6B**, *bottom panels*) were confirmed by an immunofluorescence assay. Very importantly, the cellular morphologies of autophagy induced by Sox2, including cell flattening, nuclear size and vacuole formation, were totally converted to those observed in *mock*-infected HCT116 cells (**Fig. 6B**, L.M.). Furthermore, we confirmed that the morphological recovery corresponded with restoration of Ki-67, ß-galactosidase, p16^INK4a^ and p21 protein levels in the *mock*-infected HCT116 cells (**Fig. 6C**). Notably, we confirmed that knockdown of *ATG10* in HCT116 stably expressing Sox2 recovered cell proliferation, which was inhibited by Sox2 overexpression, and the growth was even faster than that of HCT116 cells stably expressing *mock* control (**Fig. 6D**). These results indicated that ATG10 plays an important role in autophagy formation induced by Sox2 expression in HCT116 colorectal cancer cells.

### Sox2-induced Autophagy Suppresses Cancer Growth in a Xenograft Mouse Model

**Figure 7:**
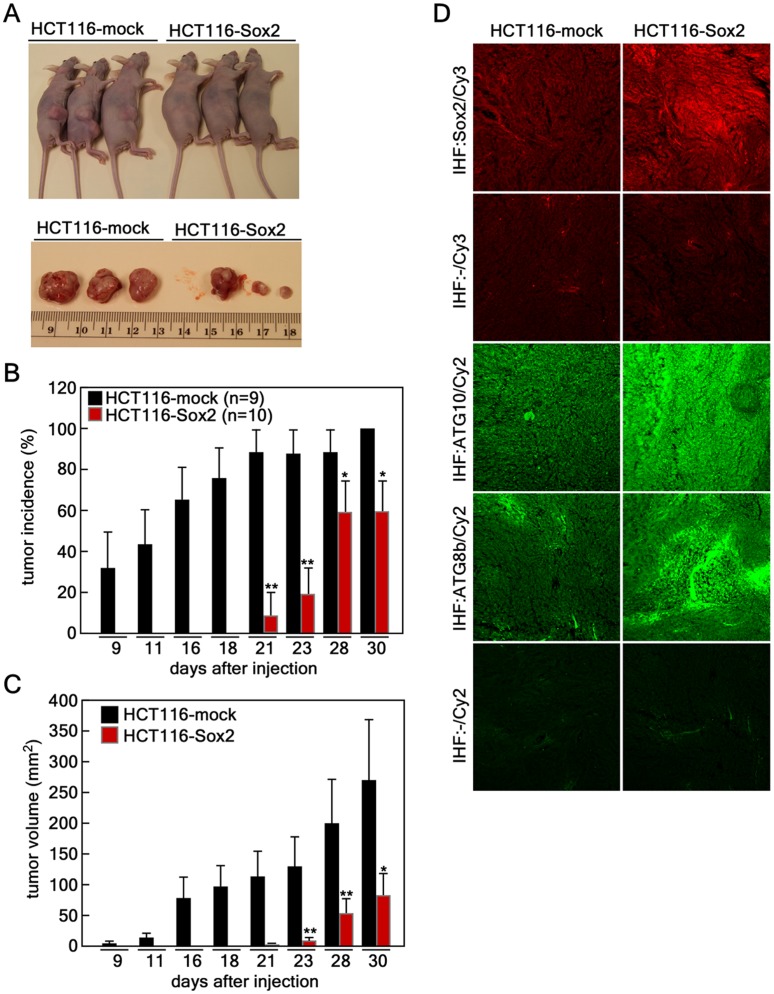
Sox2-induced autophagy suppresses cancer growth in a xenograft mouse model. (*A*) Xenograft *in vivo* tumor growth. HCT116 cells (3×10^6^) stably expressing *mock* (n = 9) or *Sox2* (n = 10) were injected subcutaneously into the dorsal right flank of athymic nude mice. At the endpoint (30 days) after injection, mice were euthanized and necropsied. (*B*) *Sox2* expression delayed tumor growth in athymic nude mice. Tumor incidence of HCT116 cells stably expressing *mock* or *Sox2* was analyzed at first measurable tumor growth after injection and compared (*p<0.05, *p<0.01). (*C*) *Sox2* expression suppresses *in vivo* tumor growth. Average tumor growth of HCT116 cells stably expressing *mock* or *Sox2* was measured and compared. Tumor volume was calculated from measurements of 2 diameters of the individual tumor according to the formula: tumor volume (mm^3^) = [longer diameter × shorter diameter^2^]/2 (*p<0.05, *p<0.01). (*D*) Immunohistofluorescence analysis of tumor tissues. The largest tissues formed from HCT116 cells stably expressing *mock* or *Sox2* were necropsied, fixed in Zamboni’s fixative and tissue slides were prepared. The tissues were hybridized with specific antibodies as indicated and visualized with Cy3 (red color) to detect Sox2 and Cy2 (green color) for detection of ATG10 and ATG8b by laser scanning confocal microscopy (X200; NIKON C1^si^ Confocal Spectral Imaging System, NIKON Instruments Co.).

Our previous results demonstrated that *Sox2*-induced autophagy suppressed cell proliferation and anchorage-independent colony growth (**Fig. 5A, B**). To determine whether Sox2 overexpression suppresses tumor growth *in vivo*, we examined xenograft tumor growth in athymic nude mice. HCT116 cells (3×10^6^) infected with *mock* or *Sox2* were injected subcutaneously into the right flank of athymic nude mice. Tumor growth was measured twice a week. At the 30-day endpoint, HCT116 cells infected with *mock* vector showed significantly larger tumor growth compared to HCT116 cells infected with *Sox2* (**Fig. 7A**). The first measurable tumor was observed at day 21 after injection of HCT116 cells infected with *Sox2* compared with day 7 in HCT116 cells infected with *mock* vector (**Fig. 7B**). Furthermore, tumor growth was markedly attenuated in athymic nude mice injected with HCT116 cells infected with *Sox2* compared with HCT116 cells infected with *mock* vector (**Fig. 7C**). To examine Sox2, ATG10 and ATG8b protein levels in tumor tissues, we conducted an immunofluorescence assay by confocal microscope using Sox2, ATG10 and ATG8b antibodies. We found that tumor tissues from HCT116 cells infected with *Sox2* displayed a higher Sox2 protein level compared with tumors from HCT116 cells infected with *mock* vector (**Fig. 7D**). Furthermore, the tumor tissues formed from HCT116 cells infected with *Sox2* exhibited higher ATG10 and ATG8b protein levels compared with tumors from the HCT116 cells infected with *mock* vector (**Fig. 7D**). Taken together, these results demonstrated that Sox2-induced autophagy suppresses cancer growth in an *in vivo* xenograft mouse model.

## Discussion

Monitoring autophagy is outlined recently in “Guidelines for the use and interpretation of assays for monitoring autophagy [34]. Although each autophagy assay is not absolutely limited to monitoring autophagy, the guidelines suggest several diverse methods, including transmission electron microscopy, Atg8-LC3 detection and quantification, SQSTM1/p62 and LC3 binding protein turnover, and assessing mTOR, AMPK and Atg1/ULK1 expression. Our results demonstrated that Sox2 overexpression induced vacuole formation and lysosomal activation (**Fig. 1**). Importantly, Sox2 induced LC3 accumulation visualized by Western blotting (**Fig. 3C**) and foci formation visualized by immunocytofluorescence (**Fig. 1D**). Moreover, our results demonstrated that the Sox2 induced *ATG10* gene expression (**Fig. 3B–F**). Thus, we hypothesized that Sox2-induced morphological and cellular functional changes are caused by autophagy.

Sox2 expression induced autophagy in all colon cancer cell lines examined. When we produced and infected *lenti-Sox2* into cells, nearly 100% of the cells were infected with *Sox2* and over 90% formed vacuoles in the cytoplasm (**Fig. 1B**). The cells exhibiting intracellular vacuoles have undergone flattening of the cytoplasm and nuclear enlargement. The Sox2 protein levels in flattened cells were lower compared with cells not flattened. Moreover, cells having small nuclei and high level of Sox2 expression also exhibited small vacuoles and continued growing (**Fig. 2C**). The population of cells undergoing cellular senescence induced by Sox2 overexpression had extensive vacuole formation (i.e., over 90%) and proliferation was actually stopped. Because of this, we conducted all experiments with new transduction each time. Importantly, ectopic expression of Sox2 induced multiple nuclei (i.e., aneuploidy), which is an important biological phenomenon that we are currently studying. (**Fig. 1D** **,** **3D** **,** **6B**).

Although recent studies have increased our understanding of the molecular mechanisms and biological functions of autophagy, whether autophagy acts fundamentally in a cell survival or cell death pathway or both is not clear. In this study, we found that autophagy inhibited cell proliferation (**Fig. 5A**) and tumor growth *ex vivo* (**Fig. 5B**) and *in vivo* (**Fig. 7A–C**). Because the role of autophagy is likened to a double-edged sword [3], a complete understanding of the role of autophagy in cancer cell growth might help in the search for clues to find strategies to treat cancer. For example, chemically-induced liver cancer cells exhibited lower autophagic activity compared with their non-neoplastic liver counterparts and transformed cell lines showed lower protein degradation rates during nutritional stress compared with nontransformed control cells [35]. Very interestingly, several chemopreventive flavonoids and other phytochemical compounds, including saponins, curcumin, genistein, quercetin, resveratrol, sulforaphane, tocotrienols and vitamins, stimulate autophagic vacuolation in many cancer cell types [2]. Furthermore, many anticancer drugs or compounds including tamoxifen, arsenic trioxide, imatinib and rapamycin induce autophagic cell death in many human cancer cell lines [10], [12], [13], [14]. Importantly, monoallelic loss of the *beclin1* gene is genetically associated with human cancers such as ovary, breast and prostate cancer [7]. Moreover, beclin-induced autophagy formation suppresses MCF-7 breast cancer cell colony growth under anchorage-independent conditions [15] and also inhibits cell proliferation by inhibition of p70 S6kinase activity [16], [17].

Many human cancers exhibit an enhanced activation of mTOR, resulting in reduced autophagy. Elegant *in vivo* experiments demonstrated that constitutively active Akt inhibits the induction of autophagy *in vitro* and *in vivo*, but accelerates the growth of tumors *in vivo*, in particular in cells that lack expression of Bax and Bak [36]. Conversely, tumor suppressor gene products upstream of mTOR signaling, such as PTEN (phosphatase and tensin homolog deleted on chromosome ten) and TCSs (tuberous sclerosis complex TSC1 and TSC2), antagonize PI3-K activation and promote autophagy by down-regulation of mTOR [37]. These studies are in agreement with our *ex vivo* results showing that ectopic expression of Sox2 reduced PTEN phosphorylation (**Fig. 4C**), resulting in subsequent reduction of phosphorylated and total Akt1 and downstream signaling molecules, such as mTOR and p70S6 kinase (**Fig. 4B**). Importantly, insulin stimulation partially rescued Sox2-induced autophagy under normal cell culture conditions (**Fig. 4D** *left panel* *s,* **E**) and also starvation conditions (**Fig. 4D** *right panel* *s,* **E**), indicating that Sox2-induced autophagy might correspond with negative regulation of Akt-mTOR signaling mediated through PTEN. Moreover, we suggest that constitutively active Akt (Myr-Akt) might rescue the autophagy formation induced by Sox2 overexpression. Although the molecular mechanism explaining how Sox2 modulates PTEN phosphorylation is not clearly understood, our results suggested that Sox2-induced autophagy might be applicable in suppressing colon cancer.

Growth arrest of senescent cells is initiated with the activation of p53 by inhibition of its degradation through the involvement of p14^ARF^, a tumor suppressor that sequesters the MDM2 protein [38]. Growth arrest can also occur through p53 acetylation mediated by the promyelocyte leukemia (PML) tumor suppressor in Ras-induced accelerated senescence [39], [40]. Cells that undergo senescence cannot divide even if stimulated by mitogens, but they remain metabolically and synthetically active and show characteristic changes in morphology, such as enlarged and flattened cell shape and increased granularity [1]. Although global and progressive epigenetic alterations are proposed to play a crucial role in oncogene-induced senescence [26], a recent study demonstrates that overexpression of ULK3, a human ortholog of ATG1, induces autophagy and senescence and inhibition of autophagy delays the senescence phenotype [8]. Our findings indicated that Sox2 overexpression induced autophagy through positive regulation of *ATG10* expression, and knockdown of *ATG10* in Sox2 overexpressing cells restored Sox2-induced autophagy and senescence phenotypes, which included vacuole formation, flattened cell morphology, and cellular senescence and proliferation markers, including ß-galactosidase, Ki-67, p16^INK4a^ and p21, to normal cell morphology (**Fig. 6A, B**). Importantly, Sox2-mediated suppression of cell proliferation was more substantially enhanced compared with parental HCT116-mock cells (**Fig. 6C**). These results demonstrated that the Sox2-ATG10 signaling pathway induces autophagy and then promotes cellular senescence. Furthermore, in our *in vivo* experiment, we found that HCT116 parental cells grew well in the xenograft mouse model (**Fig. 7A–C**). However, Sox2 overexpressing HCT116 cells grew very poorly in these mice (**Fig. 7A–C**), indicating that Sox2-induced senescence mediated through autophagy inhibits tumor growth.

Because mitotic cells cannot redistribute and eliminate damaged organelles, proteins and aggregates through cell division, basal levels of autophagy might be particularly essential for post-mitotic cells [41]. Unlike the ubiquitin-proteasome system, which is another major protein degradation system, autophagic breakdown of substrates is not only limited by steric considerations, but also uniquely capable of degrading whole organelles such as mitochondria, peroxisomes, and endoplasmic reticulum [41]. Thus, basal- and induced-autophagy are important for the physiological control of the number and quality of organelles in diverse phyla and function to eliminate superfluous, aged and damaged organelles and proteins [41]. For example, reactive oxygen species (ROS), critical inducers of autophagy, forces to initiate cardiovascular differentiation programs [42], [43], indicate that autophagy might play an important role in the ES cell self-renewal program by elimination of oxidized or damaged proteins or subcellular organelles for quality control of ES cells. Sox2 plays a key role in ES cell self-renewal and iPS production [21], [44]. The Sox2 protein level is dramatically decreased during the ES cell differentiation process [45]. Our results indicated that Sox2 overexpression induced autophagy in HCT116 colorectal cancer cells (**Fig. 1** and **2**). However, whether ES cells exhibit higher autophagic activity compared with differentiated cells is not known. In this study, we provided evidence showing that Sox2 induced autophagy through the up-regulation of *ATG10* gene expression and cellular senescence, resulting in inhibition of tumor growth *ex vivo* and *in vivo*. We also suggest that autophagy mediated by Sox2 results in loss of malignancy in colon cancer cells *ex vivo* and *in vivo.* Overall, our study could be critical to our understanding of the underlying roles of Sox2 in cancer.

## Materials and Methods

### Reagents

Chemical reagents, including Tris, NaCl, and SDS for molecular biology and buffer preparation were purchased from Sigma-Aldrich (St. Louis, MO). Restriction enzymes and some modifying enzymes were purchased from New England BioLabs, Inc. (Beverly, MA). The Taq DNA polymerase was obtained from Qiagen, Inc. (Valencia, CA). The DNA ligation kit (v. 2.0) was purchased from TAKATA Bio, Inc. (Otsu, Shiga, Japan). The lentiviral expression vectors including *pSin-EF2-Sox2, -Nanog, -Oct4* and *-Lin28* and packaging vectors including *pMD2.0G* and *psPAX* were purchased from Addgene Inc. (Cambridge, MA). Antibodies against beclin and ATG8b (LC3-b) were from ABGENT (San Diego, CA) and p16^INK4a^ and p21 were purchased from Santa Cruz Biotechnology Inc. (Santa Cruz, CA). Nanog, Oct4, Lin28 and ATG10 antibodies were purchased from Abcam (Cambridge, MA). Lysotracker was purchased from Invitrogen (Carlsbad, CA). Antibodies against Sox2, Class III PI3-K, phosphorylated p53 (Ser15), total and phosphorylated Akt, mTOR, p70 S6 kinase and ß-galactosidase were purchased from Cell Signaling Technology, Inc. (Danvers, MA). The SA-ß-galactosidase assay kit for assessing cellular senescence was purchased from Cell Signaling Technology Inc. and CellTiter 96® Aqueous One Solution for cell proliferation analysis was from Promega (Madison, WI). Dulbecco’s modified Eagle’s medium (DMEM) and McCoy’s 5a medium were purchased from Cellgro (Manassas, VA).

### Cell Culture and Transfections

HEK293T, HCT116, CCD-18Co, HT29, and WiDr cells were purchased from ATCC and maintained according to ATCC suggested conditions. Briefly, HEK293T cells were cultured with Dulbecco’s modified Eagle’s medium (DMEM) supplemented with 10% fetal bovine serum (FBS) and antibiotics at 37°C in a 5% CO_2_ incubator. HCT116 colorectal cancer cells were cultured in McCoy’s 5a medium supplemented with 10% FBS and antibiotics at 37°C in a 5% CO_2_ incubator. To prepare Sox2, Oct4, Nanog, Lin28 viral particles, each of the viral vectors and packaging vectors (*pMD2.0G* and *psPAX*) was transfected into HEK293T cells following Addgene’s suggested protocols and using JetPEI (Q-Biogene, West Chester, PA) also following the manufacturer’s suggested protocol. The transfection medium was exchanged at 4 h after transfection and then cells were cultured an additional 36 h. The viral particles were harvested by filtration using a 0.45 µm sodium acetate syringe filter and used for infection into 60% confluent HCT116 cells using 8 µg/ml of polybrane (Millipore, Billerica, MA) overnight. The cell culture medium was replaced with fresh complete growth medium and cells incubated for 24 h and then selected with 1.5 µg/ml of puromycine for 36 h.

### MTS Assay

To estimate proliferation, HCT116 cells stably expressing *mock* or *Sox2* were trypsinized, counted and then seeded into 96-well plates (2×10^3^ cells/well). After culturing for 2 h, 20 µl of the CellTiter 96® Aqueous One Solution (Promega) were added to each well and cells were then incubated for 1 h at 37°C and 5% CO_2_. To stop the reaction, 25 µl of a 10% SDS solution were added and absorbance was measured at 492 and 690 nm. Cell proliferation was measured at 24-h intervals.

### Cell Cycle Analysis

HCT116 cells (4×10^5^) stably expressing *mock* or *Sox2* were seeded into 60-mm dishes and cultured for 16 h at 37°C in a 5% CO_2_ incubator. The medium was changed and cells cultured for 24 h and then cells were trypsinized, fixed with methanol, stained with propidium iodide (PI) and then analyzed for cell cycle phase using the FACS Calibur (Becton Dickinson, Franklin Lakes, NJ).

### Western Blotting

Proteins were extracted with RIPA cell lysis buffer by freezing and thawing and protein concentration was measured. The same amount of protein was resolved by SDS-polyacrylamide gel electrophoresis, transferred onto PVDF membranes and blocked in 5% skim milk for 1 h at room temperature. Antibody hybridization was performed overnight at 4°C and HRP-conjugated secondary antibody binding was then conducted for 1 h at room temperature. The membranes were washed and proteins visualized using the ECL detection kit (Amersham Biosciences, Piscataway, NJ).

### cDNA Microarray

The total RNAs from HCT116 cells stably expressing *mock* or *Sox2* were extracted using an RNA extraction kit (Qiagen, Valencia, CA). The RNA purity for this experiment was over 2.0 in the ratio of OD^260^/OD^280^. The microarray was conducted by the Phalanx Biotech Group (Palo Alto, CA) and duplicate array results were obtained using the Significance Analysis of Microarrays (SAM) program from the Stanford Microarray database (http://smd.stanford.edu). SAM identified the genes with statistically significant changes in expression by performing a set of gene-specific *t-*tests (p<0.05). The genes were color-coded as red for up-regulation and green for down-regulation. The identified genes were annotated using the DAVID database (http://niaid.abcc.ncifcrf.gov) [46], [47].

### Immunofluorescence Assay

HCT116 cells stably expressing *mock*, *Sox2* or *shATG10* were seeded in 2- or 4-chamber slides depending on the experiment and cultured 24 h. The cells were fixed with 4% formalin, permeabilized with 0.5% Triton X-100/1X PBS for 10 min, hybridized with anti-Sox2, ATG10 or ATG8b as a primary antibody and anti-mouse IgG goat-conjugated with Alexa 568 or Alexa 488 (Invitrogen) as a secondary antibody. The cells were mounted with Fluoro-GEL II containing DAPI (Electron Microscopy Sciences, Hatfield, PA) and observed under a fluorescence microscope (X200). Tumors were excised and dropped-fixed into Zamboni’s fixative [0.03% picric acid (w/v) and 2% paraformaldehyde (w/v)] for 48 h at 4°C and then transferred to a 20% sucrose solution with 0.05% sodium azide in phosphate buffered saline (PBS) for storage. Processing and staining of tumors were carried out according to a published procedure [48]. Optical sections were captured by laser scanning confocal microscopy (NIKON C1si Confocal Spectral Imaging System, NIKON Instruments Co., Melville, NY) [48].

### Anchorage-independent Colony Assay

Anchorage-independent colony growth was investigated using HCT116 cells stably expressing *mock* or *Sox2*. In brief, cells (8×10^3^/ml) were mixed in 1 ml of 0.3% McCoy’s 5a agar containing 10% FBS. The cultures were maintained in a 37°C, 5% CO_2_ incubator for 5–7 days and the cell colonies were scored using a microscope and the Image-Pro PLUS (v.6) computer software program (Media Cybernetics, Silver Spring, MD) as described by Colburn *et al.* [49].

### *In vivo* Tumor Growth Assay

HCT116 cells stably expressing *mock* or *Sox2* were harvested at 90% confluence and suspended (3×10^6^) in 200 µl of McCoy’s 5a medium without FBS or antibiotics. Mice were divided into 2 groups of 9 or 10 mice each. Each individual cell line (3×10^6^) was injected into mice (9, *mock* vector control; 10, *Sox2*). The cells were injected subcutaneously into the right flank of each mouse. The mice were weighed twice each week and monitored every day for tumor growth. When tumors appeared, they were measured twice a week by computerized caliper. Tumor volume was calculated from measurements of 2 diameters of the individual tumor according to the formula: tumor volume (mm^3^) = [longer diameter × shorter diameter^2^]/2. Mice were monitored until tumors reached 1,000 mm^3^ total volume at which time mice were euthanized and tumors extracted for histochemical analysis. All experiments were performed according to guidelines approved by the University of Minnesota Institutional Animal Care and Use Committee.

### Ex vivo and Xenograft Animal Studies

The human cells, including HEK293T, HCT116, CCD-18Co, HT-29, and WiDr cells, used in *ex vivo* studies were purchased from ATCC and studies were approved by the University of Minnesota Institutional Biosafety Committee (IBC). The xenograft animal study utilizing athymic nude mice was conducted under the approval of the University of Minnesota Institutional Animal Care and Use Committee (IACUC).

### Statistical Analysis

Each experiment was conducted at least two times independently in triplicate. Data are shown as means ± S.D. (standard deviation) or S.E. (standard error) of values obtained from triplicate experiments as indicated in each figure legend. Significant differences were evaluated using the Student’s *t*–test.

## Supporting Information

Table S1 **Microarray of HCT116 colorectal cancer cells stably expressing** ***Sox2*** **.** These microarray results were compared with HCT116 cells stably expressing a *mock* vector control. The red color indicates up-regulated genes, green color denotes down-regulated genes, and the yellow color indicates no difference in gene expression.
(ZIP)

Table S2 T**he microarray results are grouped as indicated using the DAVID Database (** http://niaid.abcc.ncifcrf.gov **) (1, 2) and the numbers of genes up- (red) or down-regulated (green) are denoted.** The *p* value was obtained by *t-*tests comparing gene expression in HCT116 cells expressing *Sox2* with HCT116 cells expressing *mock* control.
(ZIP)

Table S3 **Summary of gene expression in cellular responsiveness to** ***Sox2*** **expression.** Table S3 summarizes gene expression profiles (from **Table S2**) in HCT116 colorectal cancer cells induced by *Sox2* expression compared with *mock* expression.
(TIF)

Table S4 **Identity of putative consensus sequences in autophagy-related genes.** The data were obtained by a re-analysis of results from a previous publication (3) and summarized. The *Sox2-* associated score indicates the identity of Sox-binding consensus sequences, 5′-CATTGAT-3′ (4).
(TIF)

References S1 (DOCX)
