# Supplementary figures and images for: Autophagy and Cellular Senescence Mediated by Sox2 Suppress Malignancy of Cancer Cells
Source: PLoS One. 2013 Feb 25;8(2):e57172. doi: 10.1371/journal.pone.0057172 (PMC3581442; doi:10.1371/journal.pone.0057172)

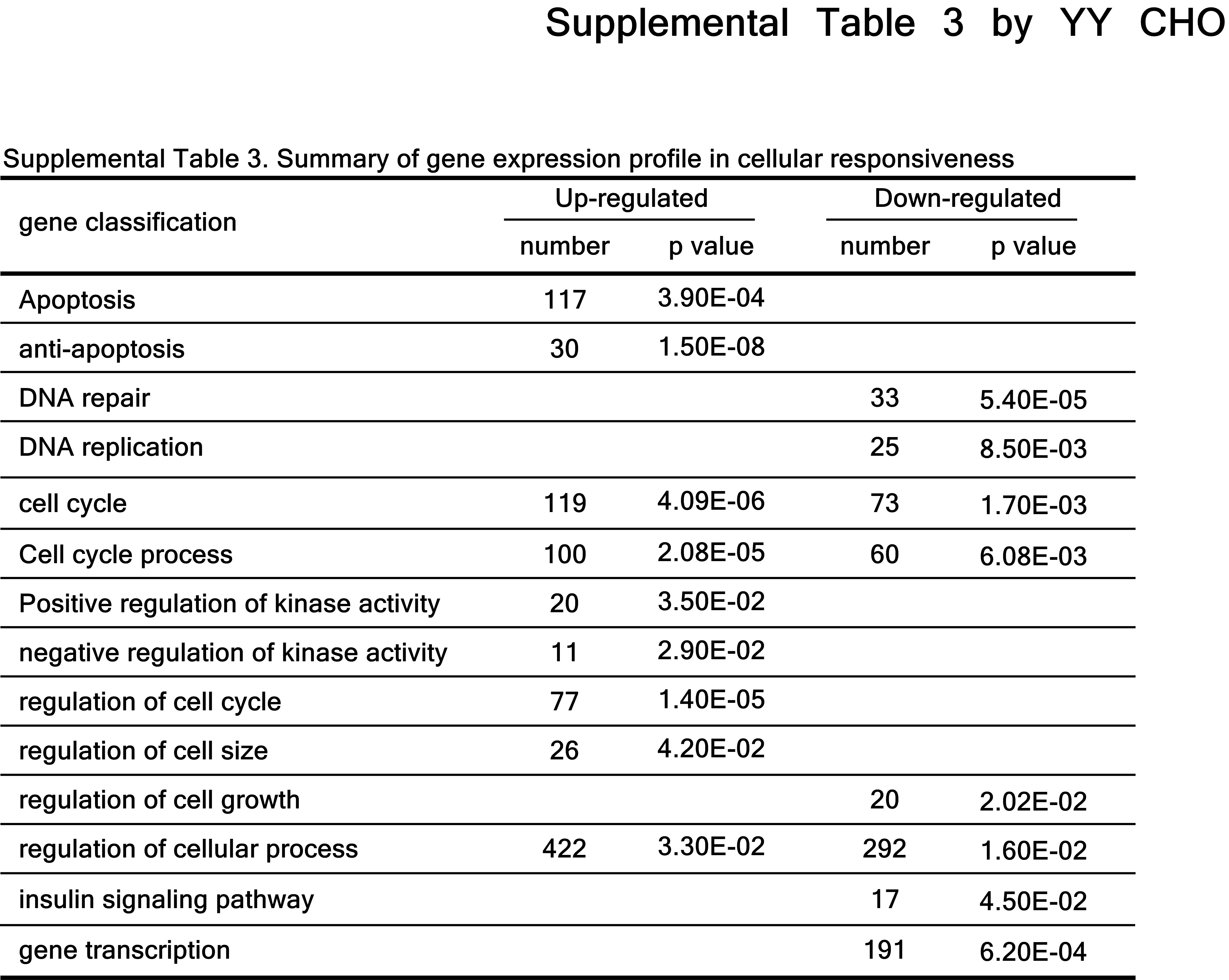

Supplement: Table S3 — Summary of gene expression in cellular responsiveness to Sox2 expression. Table S3 summarizes gene expression profiles (from Table S2) in HCT116 colorectal cancer cells induced by Sox2 expression compared with mock expression. (TIF) [file pone.0057172.s003.tif]

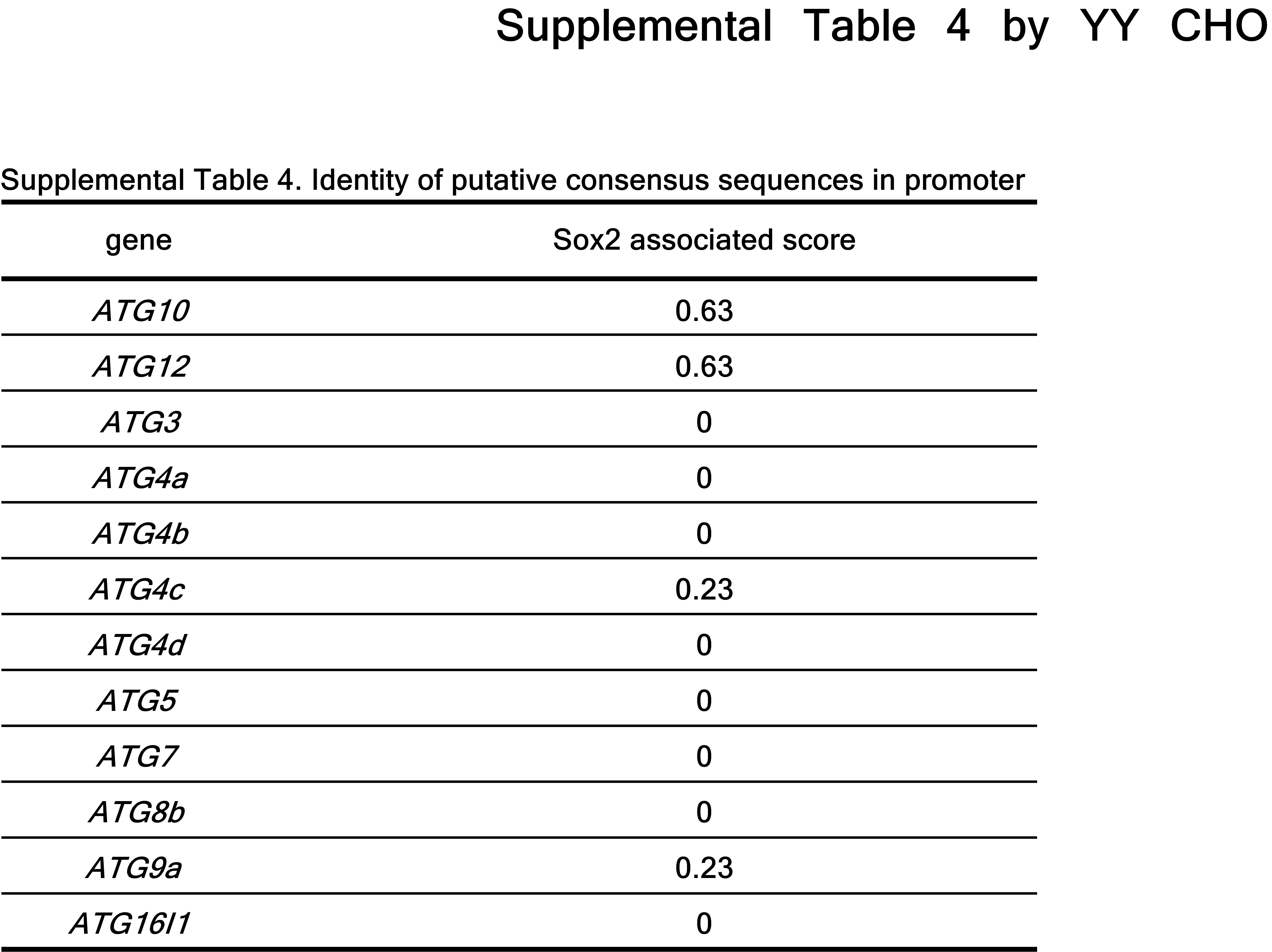

Supplement: Table S4 — Identity of putative consensus sequences in autophagy-related genes. The data were obtained by a re-analysis of results from a previous publication (3) and summarized. The Sox2- associated score indicates the identity of Sox-binding consensus sequences, 5′-CATTGAT-3′ (4). (TIF) [file pone.0057172.s004.tif]
