# Supplementary material for: Autophagy and Cellular Senescence Mediated by Sox2 Suppress Malignancy of Cancer Cells
Source: PLoS One. 2013 Feb 25;8(2):e57172. doi: 10.1371/journal.pone.0057172 (PMC3581442; doi:10.1371/journal.pone.0057172)
Supplement: References S1 — (DOCX) [file pone.0057172.s005.docx]

1. **SUPPLEMENTAL REFERENCES**

1. Huang da, W., Sherman, B.T., and Lempicki, R.A. 2009. Systematic and integrative analysis of large gene lists using DAVID bioinformatics resources. *Nat Protoc* 4:44-57.

2. Dennis, G., Jr., Sherman, B.T., Hosack, D.A., Yang, J., Gao, W., Lane, H.C., and Lempicki, R.A. 2003. DAVID: Database for Annotation, Visualization, and Integrated Discovery. *Genome Biol* 4:P3.

3. Chen, X., Xu, H., Yuan, P., Fang, F., Huss, M., Vega, V.B., Wong, E., Orlov, Y.L., Zhang, W., Jiang, J., et al. 2008. Integration of external signaling pathways with the core transcriptional network in embryonic stem cells. *Cell* 133:1106-1117.

4. Loh, Y.H., Wu, Q., Chew, J.L., Vega, V.B., Zhang, W., Chen, X., Bourque, G., George, J., Leong, B., Liu, J., et al. 2006. The Oct4 and Nanog transcription network regulates pluripotency in mouse embryonic stem cells. *Nat Genet* 38:431-440.
